# Supplementary figures and images for: Segregational drift hinders the evolution of antibiotic resistance on polyploid replicons
Source: PLoS Genet. 2023 Aug 3;19(8):e1010829. doi: 10.1371/journal.pgen.1010829 (PMC10399855; doi:10.1371/journal.pgen.1010829)

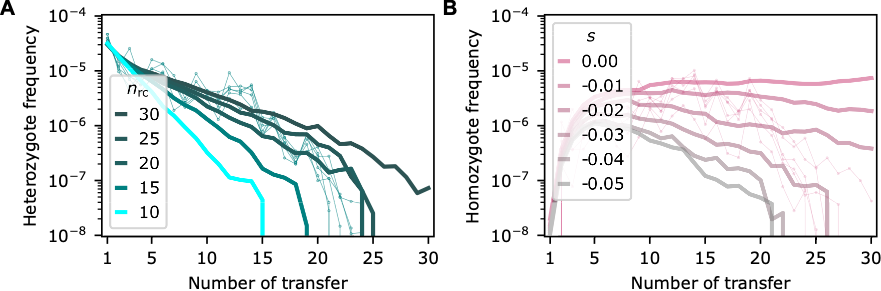

Supplement: S1 Fig — Simulations with (A) different replicon copy numbers nrc (see legend) under non-selective conditions (s = 0) and with (B) nrc = 15 and different negative selection parameters s<0. Simulations and experiments were performed with high initial frequencies, f0 = 10−4, and weak bottlenecks, b = 10−2. Bold green (red) lines show the median of the heterozygote (homozygote) frequencies before each transfer from 10 simulations for each replicon copy number. Thin lines show the heterozygote frequency from the six replicates of the evolution experiment. Simulation results were obtained as described for Fig 1D. (TIF) [file pgen.1010829.s001.tif]

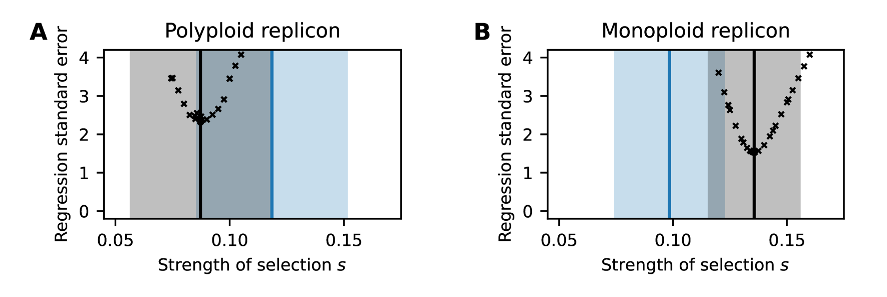

Supplement: S8 Fig — a) The regression standard error, χν2=χ2ν where χ2=∑B=130eB2 (weighted sum of squared deviations) and ν = 30−1 (degree of freedom), for the simulated allele dynamics on the polyploid replicon (nrc = 15) with different selection coefficients s, which yields an optimal fit to the experimental allele dynamics on the chromosome with s = 0.087 ± 0.016 (standard error). b) The simulated allele dynamics on the monoploid replicon (nrc = 1), which yields an optimal fit to the experimental allele dynamics on the multicopy plasmid with s = 0.136 ± 0.010. We fitted the selection coefficients using the cell frequencies of the novel phenotype (kanamycin-resistant) for the experimental conditions f0 = 10−4 and b = 0.01. Blue lines show the experimentally estimated selection coefficients. The minimization of the residuals was initiated at s = 0.1 using standard least-square minimization methods (see text). The plots above show data points for the regression standard error additional to the s values reached by the minimization method. The boxes show the 95% confidence intervals of the estimated s values for the experimental s (blue) and the theoretical s (gray). (TIFF) [file pgen.1010829.s008.tiff]

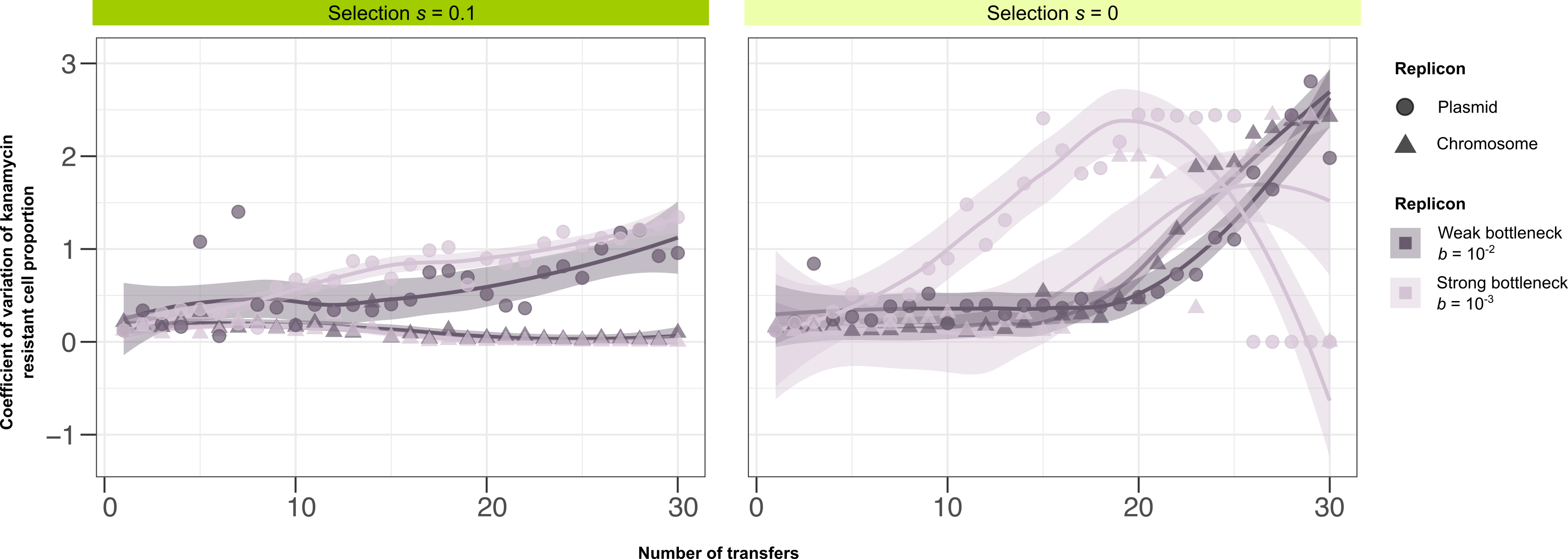

Supplement: S9 Fig — Lines present the trendline and the shaded area corresponds to the confidence interval. (TIFF) [file pgen.1010829.s009.tiff]

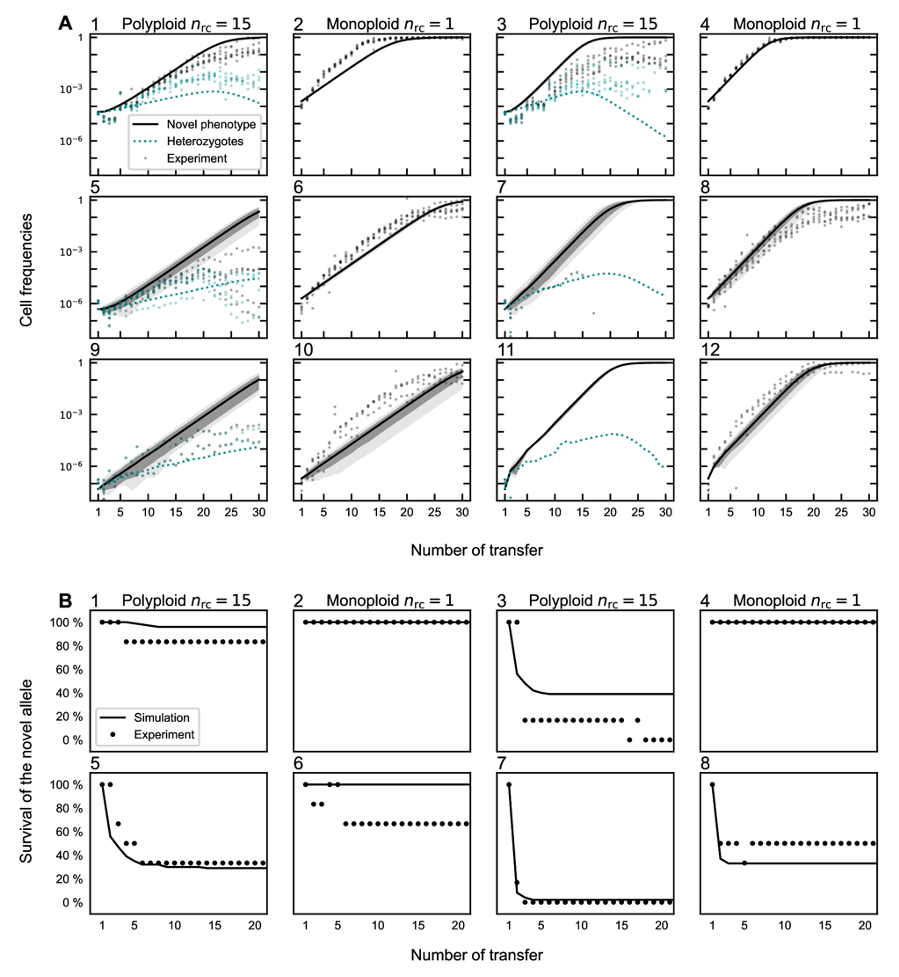

Supplement: S10 Fig — Analogue to Fig 3, where we used s = 0.087 (s = 0.136) for the novel allele on the polyploid (monoploid) replicon, predicted frequencies of the novel phenotype are shown in (A) and the corresponding survival of the novel allele are shown in (B). (A) Cell frequencies of the novel phenotype (parallel to kanamycin-resistant cells) (black) and heterozygous cells (green) at the end of each population transfer obtained from 100 simulations for a replicon with copy number nrc = 15 (1,3,5,7,9,11; for the multicopy plasmid) and with nrc = 1 (2,4,6,8,10,12; reflecting the chromosome). Lines show the mean of positive frequencies over the number of transfers, i.e., the average proportion of cells having the novel phenotype from all simulation trajectories, where the novel allele has not (yet) gone extinct (cf. (B) for the survival of the allele). The initial population at the start of the first transfer consists of ancestral cells and a small proportion, f, of cells with one novel replicon copy, where f0 = 10−4 in (1–4), f0 = 10−6 in (5–8), and f0 = 10−7 in (8–12). The grey (dark grey) areas show the 99% (67%) confidence intervals of positive frequencies of the novel phenotype. The green lines show the mean frequency of heterozygous cells carrying both the ancestral and novel plasmid allele (conditioned on positive frequencies). Grey and green markers show the frequencies of kanamycin-resistant cells and heterozygous cells, respectively, of the corresponding experimental replicates. (B) Survival of the novel allele over number of transfers in model simulations (lines) and in the evolution experiment (markers) for intermediate initial frequencies, f0 = 10–6, (1–4) and low initial frequencies, f0 = 10–7. The survival of the novel allele initially present at high initial frequencies, f0 = 10–4, are not shown since it is always 100%. The fraction of kanamycin-resistant cells of 100 simulation trajectories and 6 experimental replicates, respectively, is shown for each parameter comb [file pgen.1010829.s010.tiff]
